# COVID-19 pandemic and its impact on dental education: digitalization – progress or regress? Example of an online hands-on course

**DOI:** 10.1186/s12909-022-03638-7

**Published:** 2022-08-01

**Authors:** Nicolai Oetter, Tobias Möst, Manuel Weber, Mayte Buchbender, Maximilian Rohde, Yannick Foerster, Charlotte Bauerschmitz, Nico Röschmann, Werner Adler, Andrea Rau, Marion Meyerolbersleben, Marco Kesting, Rainer Lutz

**Affiliations:** 1 Department of Oral and Cranio-Maxillofacial Surgery, Friedrich‑Alexander-Universität Erlangen‑Nürnberg (FAU), University Hospital Erlangen, Glückstraße 11, 91054 Erlangen, Germany; 2 Department of Biometry and Epidemiology, Friedrich-Alexander-Universität Erlangen-Nürnberg (FAU), Waldstraße 6, 91054 Erlangen, Germany; 3 Department of Oral and Cranio-Maxillofacial Surgery, University of Greifswald, University Hospital Greifswald, Ferdinand-Sauerbruch-Straße DZ 7, 17475 Greifswald, Germany; 4 Institute for Innovation in Learning, Friedrich‑Alexander-Universität Erlangen‑Nürnberg (FAU), Dr.-Mack-Straße 77, 90762 Fürth, Germany

**Keywords:** Education, Dentistry, Coronavirus, Pandemic, Digital, Asynchronous, Synchronous

## Abstract

**Background:**

Due to the SARS-CoV-2 pandemic and the accompanying contact restrictions, a new challenge arose for dental education. Despite the limited overall situation, it must be ensured that, in addition to theoretical content, practical skills in particular continue to be taught. Therefore, the aim of this study was to develop and implement an online hands-on course for dental students that ensures practical training, even during the pandemic.

**Methods:**

The newly developed course was held from April 2020 to March 2021. A total of six groups (each consisting of approximately 40–50 students) took part in the course. The participating students were in their 3rd, 4th or 5th year of study. The course taught theoretical basics (via an online platform) and promoted the learning of practical/surgical techniques on models such as bananas, pork bellies, or chicken thighs with live demonstrations (via ZOOM) and interactive post-preparation by students at home (and in a rotating small group of 3–7 students on site). Student self-evaluation (at the beginning and end of the course) and course evaluation were performed using questionnaires. The learning success was analyzed (through self-evaluations) using Wilcoxon signed-rank tests (significance level alpha = 0.05).

**Results:**

Concerning students´ self-evaluations, the theoretical knowledge, general surgical skills (such as surgical instrument handling), and specific surgical skills (such as performing a kite flap) improved during the course, with significant results (*p* < 0.001 for each). About 60% of the students rated the course overall as excellent (grades 9 or 10 on a Likert scale of 1 to 10). The technical implementation of the course was rated with a median of 9 (= very good, on a Likert scale of 1 to 10). 38.5% described the applicability of the skills learned for their later professional life as extremely good.

**Conclusions:**

The results of this work suggest that, within the limitations of this study, the introduced concept of an online hands-on course could be an appropriate form of teaching practical dental skills, even during a pandemic. Further research is needed in the field of digital education for dental students.

## Background

The pneumonia caused by the SARS CoV-2 virus (and its concomitant complications) referred to the World Health Organization (WHO) as "Coronavirus Disease 2019 (COVID-19)" has been one of the greatest public health challenges in the modern world since late 2019 [1, 2]. The coronavirus outbreak was declared an international public health emergency by the WHO on Jan. 30, 2020 [3]; the social and medical consequences are still being felt today.

Since the exact transmission routes of the coronavirus were not known at the beginning of the pandemic, the personal protective equipment of medical and dental professionals was initially assigned an enormously important role. This protective barrier equipment is primarily understood to mean protective goggles, masks, gloves, caps, face shields, and adequate protective clothing, which make patient treatment very costly and time-consuming. In addition, it was recommended that a patient with COVID-19 infection who is in the acute febrile phase of the disease should not be treated dentally, or only in an emergency [4]. For example, uncontrolled bleeding or diffuse soft tissue swelling potentially affecting the patient´s airway have been described as emergencies [5].

Currently, it is known that the transmission of the pathogen occurs either by direct transmission, such as inhalation of virus-containing droplets/microdroplets (by coughing or nosing of the patient or aerosols generated by dental procedures) or by contact with oral, nasal, and ocular mucosa (contact transmission) [4, 6]. Preliminary studies have shown that in dental procedures such as the use of high-speed handpieces with 400,000 rpm (e.g., for the removal of carious tooth lesions), the surgical removal of bone parts of the jaw with a rotating ball thread and necessary water cooling, or the use of ultrasonic scalers, a large amount of contaminated droplets and aerosols are produced, which may be mixed with the saliva and blood of the patient and remain in the air for a longer period of time before they settle on surfaces in the environment or enter the respiratory tract of the dentist [7–10]. Due to these specific dental procedures and the fact that dental practitioners cannot always respect the interpersonal distance recommended (with the patients´ mouth as the working area), there is a significantly increased risk of infection. In an article published by the New York Times (March 2020), the dental profession was labeled with the highest risk of infection among all healthcare professions [11].

Of course, dental students are also exposed to such aerosols during their clinical training (on patients), and thus to an increased risk of infection. Prior to the COVID outbreak, the Department of Oral and Cranio-Maxillofacial Surgery Erlangen-Nürnberg offered practical clinical courses for dental students (besides theoretical teachings) that were conducted separately for each clinical semester (semester 6–10). In particular, this means that students from the 6th semester onwards (from the 3rd year of training) also had to attend a clinical course in each case parallel to the lectures (theory). These courses were a mandatory part of the undergraduate program. The duration of the course was 1–2 weeks per semester, whereby different skills (such as infiltration or block anesthesia, e.g. inferior alveolar nerve block) were to be acquired. Additionally, these “face-to-face” courses included, for example, surgical assistance on patients and training on mannequins (e.g., suture practice). Along with the spreading of the coronavirus in Germany (01–03/2020) and the following lockdown, which included broad contact limitations, even dental/medical institutes closed their doors for students. At this point in time, all educational and research activities had to be stopped suddenly. To be able to continue not only theoretical but also practical teaching, the Department of Oral and Cranio-Maxillofacial Surgery of Erlangen-Nürnberg decided to implement an online hands-on course called “SOS Course: Surgical Online Skills.” Additionally, the Friedrich-Alexander-Universität Erlangen-Nürnberg initiated a support program to ensure that even students with low financial backgrounds had access to all online learning services.

The aim of this course implementation was to continue teaching practical skills to dental students even in pandemic times and to use these first results for discussing the potential of digital teaching concepts in dentistry.

## Methods

### Teaching concept and course structure

The SOS (“Surgical Online Skills”) Course was implemented as a curricular seminar in the summer term 2020 (April to September 2020) and continued during the following winter term (October 2020 to March 2021). Only students who had completed the pre-clinical section of dental school and were consequently allowed to carry out further training on patients had to participate in the course. In the period from April 2020 to March 2021, a total of six groups, each consisting of approximately 40–50 students, took part in the course. The participating students were in their 3rd, 4th or 5th year of study. For each student, participation took place over a period of one semester, which consisted of 13 live online sessions (see Table 1).

**Table 1:** Overview of the course content and timetable (over the period of one semester)

**Sessions** **(on a weekly basis)**	**Content**	**Materials required** surgical equipment & suture material +
1	Introduction, timetable, theoretical and practical basics (handling of surgical equipment)	-
2	Clinical Examination I (CE): Single button suture & horizontal mattress suture	banana, pork belly
Self-evaluation I (questionnaire)		
3	Repetition theoretical and practical basics & Single button suture	banana
4	Repetition single button suture & Continuous sutures	banana
5	Repetition continuous sutures & Horizontal and vertical mattress suture	banana, pork belly
6	Repetition mattress sutures & Z-plasty (30 and 60 degree)	pork belly
7	Repetition Z-plasty (60 degree) & Kite Flap	pork belly
8	Repetition Z-plasty (30 degree) & H-Flap	pork belly
9	Repetition H-Flap & Full thickness skin graft (incl. thinning)	pork belly, chicken thighs
10	Free mucosal transplant & Tooth extraction (treatment under anticoagulation therapy)	pig head (half)
11	Repetition tooth extraction (treatment under anticoagulation therapy) & Mucoperiosteal Flap	pig head (half)
12	Repetition full thickness skin graft (incl. thinning) & Defect covering (suturing on pork belly)	pork belly, chicken thighs
13	Clinical Examination II (CE): Tooth extraction & Mucoperiosteal Flap	pig head (half)
Self-evaluation II (questionnaire) & Course evaluation		

**Fig. 1:**
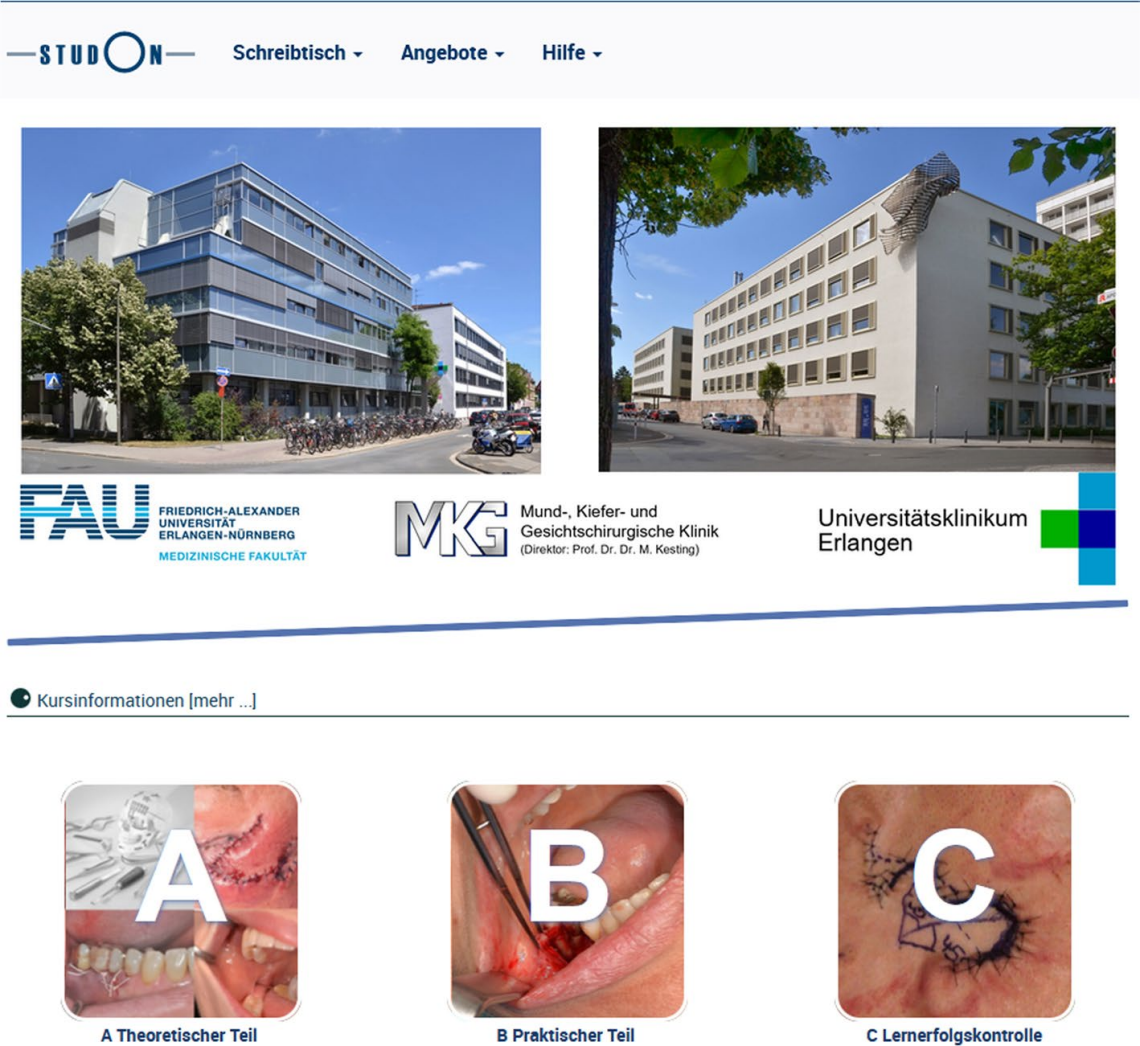
Modular course structure of the SOS Course. Module **A**: Theoretical part; Module **B**: Practical part with live online sessions; Module **C**: Learning outcome

The SOS Course has a modular structure that is based on an online teaching platform called StudOn (Friedrich-Alexander-Universität Erlangen-Nürnberg, FAU, Erlangen, Germany; realized with ILIAS 5.4.17 and Institut für Lern-Innovation, ILI, Fürth, Germany) with synchronous (live sessions) and asynchronous (theoretical) content. The course contains three modules (see Fig. 1):

#### Module A

**Fig. 2:**
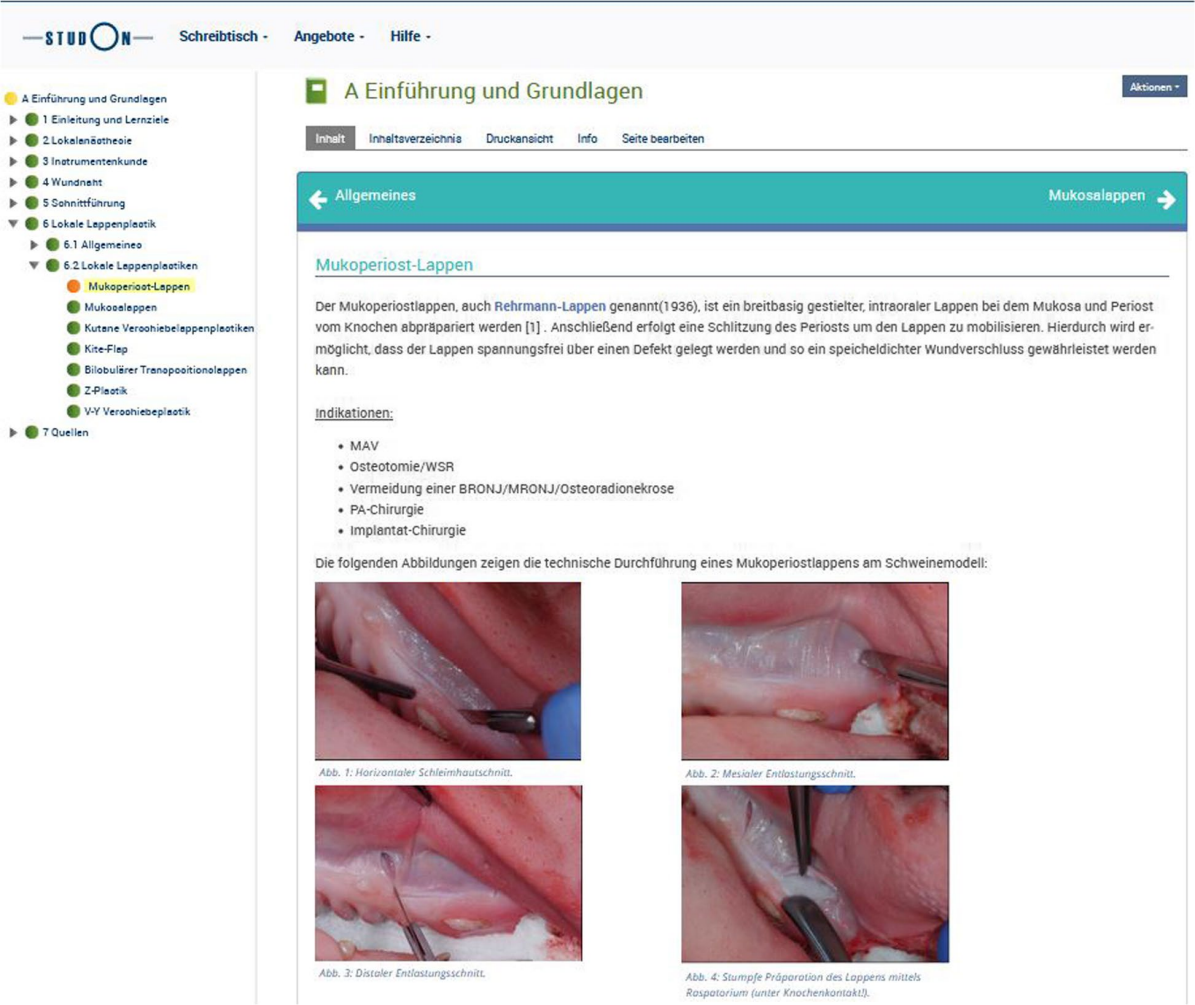
Example view of Module A (theoretical part). Interactive interface for self-study (e.g., local anesthesia, wound management, surgical incision techniques, and basics of local pedicled and free flaps) based on an online teaching platform called StudOn (Friedrich-Alexander-Universität Erlangen-Nürnberg, FAU, Erlangen, Germany; realized with ILIAS 5.4.17 and Institut für Lern-Innovation, ILI, Fürth, Germany)

The first module is a theoretical part within an interactive interface for self-study (including, e.g., tutorial videos, drag-and-drop tasks, and multiple-choice questions). This module contains information for students about course structure, learning objectives, and materials required as well as all theoretical information concerning the content of the course, e.g., local anesthesia, wound management, surgical incision techniques, and basics of local pedicled and free flaps (see Fig. 2).

#### Module B

**Fig. 3:**
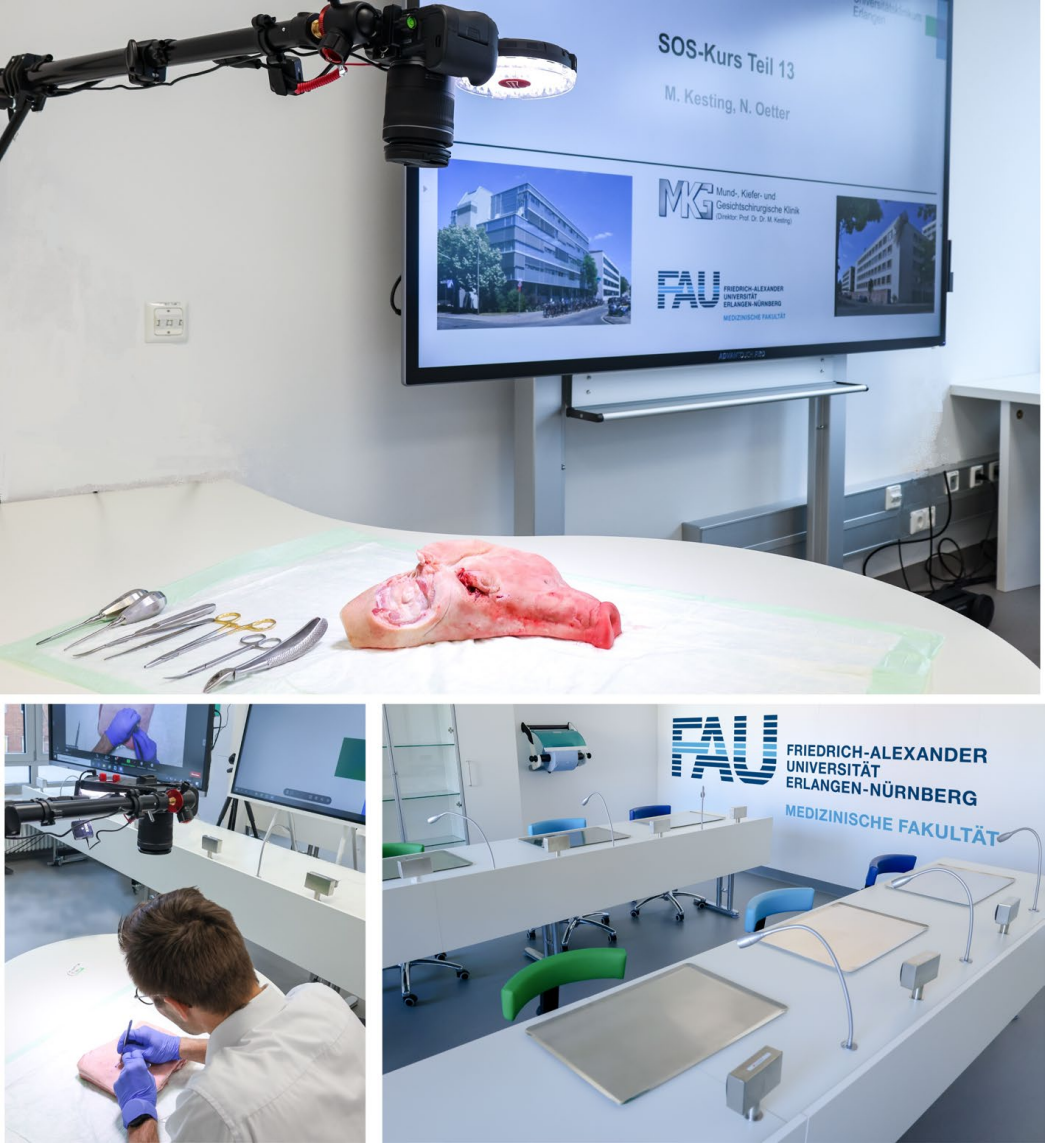
“Skills Lab” of the Department of Oral and Cranio-Maxillofacial Surgery, Friedrich‑Alexander-Universität Erlangen‑Nürnberg (FAU). Premises and technical equipment/setup for executing the SOS Course

The practical part is performed during multiple live online sessions (a total of 13 sessions). It was held for one teaching session per week (every Monday afternoon), with a duration of about 2.5 h. Therefore, the online videoconference system called ZOOM (Zoom Video Communications, Inc.; San José, California, USA; 2011) was used. Every single live session was held in our “skills lab” (see Fig. 3) and had a preset structure:

1. Introductory lecture (10–20 min.): Repetition of previous content, presentation of new content, and educational goals (short theoretical part using PowerPoint presentations).
2. Demonstration of surgical techniques (60–100 min.*):* Live preparations using appropriate models (e.g., bananas, pig heads, pork belly, or chicken thighs). Lecturers show and describe in detail how to perform surgical techniques, e.g., suturing techniques or local flap techniques (see complete course content in Table 1).
  All participating students are online at the same time (always Monday afternoon) and connected via ZOOM. Through live camera transmission, they are able to follow live preparations and perform the tasks at home without any contact with other students and consequently without any risk of infection. It must be mentioned that there is a (very small) group of students who attend in person. The reason for this is that these students were already in the clinic due to other university commitments. To prevent missing the course day, the affected students can attend the course in our skills lab. There are a maximum of 3 –7 students who change weekly. Hereby, safety measures (such as isolation distances) can be assured. This small group will be neglected in our study.
  During the sessions, lecturers and tutors give practical advice and are available for requests (online via ZOOM as well as physically in the skills lab); they promote interactivity and support discussions during the sessions (via chat or voice). During each course day, two tutors and one to two lecturers were present. The ratio of online students to tutors/lecturers was thus 23:1 (in the winter term) and 45:1 (in the summer term).
3. Concluding discussion and outlook on the next session (including necessary materials).

#### Module C

Photo documentation of the students´ practical results. As proof of participation, each student must document (via photo) the achievements of their practical exercises. Subsequently, these images have to be uploaded to StudOn at the end of each session (within Module C).

### Students´ evaluation/feedback

#### Self-evaluation: Questionnaires (via StudOn)

All students had to participate in two “Clinical Examinations” (CEs). The first was scheduled at the beginning, the second at the end of the semester/of the SOS Course (in particular, during sessions 2 and 13; see Table 1).

The "CEs" are not like classic “Objective Structured Clinical Examinations” (OSCEs) in presence (like before the pandemic) where the entire examination is objective and highly structured and in which a score is awarded. It is a clinical task in which each student has the same task objectively. This task is to be fulfilled by the students in a structured manner and in the best possible way.

Both CEs were followed by a self-evaluating questionnaire (see Table 2) to define a starting point and endpoint with regard to the student´s practical skills. The questions should support the participant´s self-reflection and yield conclusions about the current state of practical training and the overall learning success. A response option to abstain (”I don’t know”) was added to the questionnaire to prevent/reduce final bias in the results.

**Table 2:** Questionnaire on self-assessment of the students´ surgical skills (during sessions 2 and 13)

1. How do you assess your general surgical competences? *by means of German school grades (1* = *very good to 6* = *insufficient; 7* = *I don´t know)*									
Handling of surgical instruments (Q1)									
Manual surgical skills (Q2)									
Theoretical skills (Q3)									
2. How do you assess your specific surgical competences? *by means of German school grades (1* = *very good to 6* = *insufficient; 7* = *I don´t know)*									
Ability to coach and direct others (Q4)									
Suturing techniques (Q5)									
Z-Plasty (Q6)									
Kite-Flap (Q7)									
H-Flap (Q8)									
Full thickness skin graft (removal and processing) (Q9)									
Free mucosal transplant (Q10)									
Tooth extraction (Q11)									
Mucoperiosteal Flap (Q12)									
3. For each of the subsequent listed statements, please indicate the extent to which it applies or does not apply:									
	I absolutely agree	I somewhat agree	I rather disagree			I do not agree at all			I don´t know
I am convinced that I will be able to cope well with the future practical requirements (during the course/in working life). (Q13)	❍	❍	❍			❍			❍
I am rather relaxed about possible difficulties during the practical work because I can trust in my own capabilities. (Q14)	❍	❍	❍			❍			❍
Others are better able to cope with future practical requirements (during the course/in working life). (Q15)	❍	❍	❍			❍			❍

Self-estimation of practical surgical skills and abilities is conducted at two different points in time: 1. At the beginning of the SOS Course (session 2). 2. At the end of the SOS Course (session 13)

#### Course evaluation: Questionnaire (via StudOn)

At the end of the semester, there was an evaluation session to assess the course in terms of technical implementation, course content, temporal organization, and student support. Data collection was conducted anonymously and satisfied the demands of data protection and privacy policy. All course participants were informed that the course evaluation had no impact on passing the course. The evaluation could be done after the end of the course, i.e., after the last session. The students had a period of four weeks (a period of reflection) until the evaluation session was closed.

For course evaluation, an online questionnaire was used via StudOn (Friedrich-Alexander-Universität Erlangen-Nürnberg, FAU, Erlangen, Germany; realized with ILIAS 5.4.17 and Institut für Lern-Innovation, ILI, Fürth, Germany) that included matrix questions (anonymous and encrypted).

### Statistics

#### Inclusion criteria

- ◦ All questionnaires in which more than 90% of the questions were answered by the students were included in the statistical analysis.

#### Exclusion criteria

- ◦ Questionnaires were excluded from statistical analysis if ≥ 10% of the questions were not recorded/not answered by the students.
- ◦ In addition, questionnaires were excluded when ≥ 90% of the questions were answered with the same answer choice (e.g., answer choice 1 in 100% of the questions). It is assumed that these questionnaires were answered with the same answer option to save time and to complete the evaluation as quickly as possible. These non-truthful responses with the student goal of expeditious evaluation completion would skew the statistical analysis.

In the aforementioned cases, the remaining answered questions were also not taken into account, and thus the entire questionnaire was excluded.

The statistical analysis was performed with IBM SPSS Statistics 24 (Released 2016. IBM SPSS Statistics for Windows, Version 24.0. Armonk, NY: IBM Corp.). In the presence of non-normally distributed data, the usual parameters were calculated to determine the positional measures and dispersion parameters (descriptive statistics). The statistical comparison of the practical skills of the students before (self-evaluation 1; SE1) and after (self-evaluation 2; SE2) implementation of the newly introduced SOS Course was performed. Likewise, the learning success of the students was evaluated with regard to the increase in self-assessed theoretical knowledge (comparison of self-assessed theoretical knowledge before/after the SOS Course). In both cases, the Wilcoxon signed rank test (significance level alpha = 0.05) was applied to test for differences in location.

## Results

### Practical results of the students

**Fig. 4:**
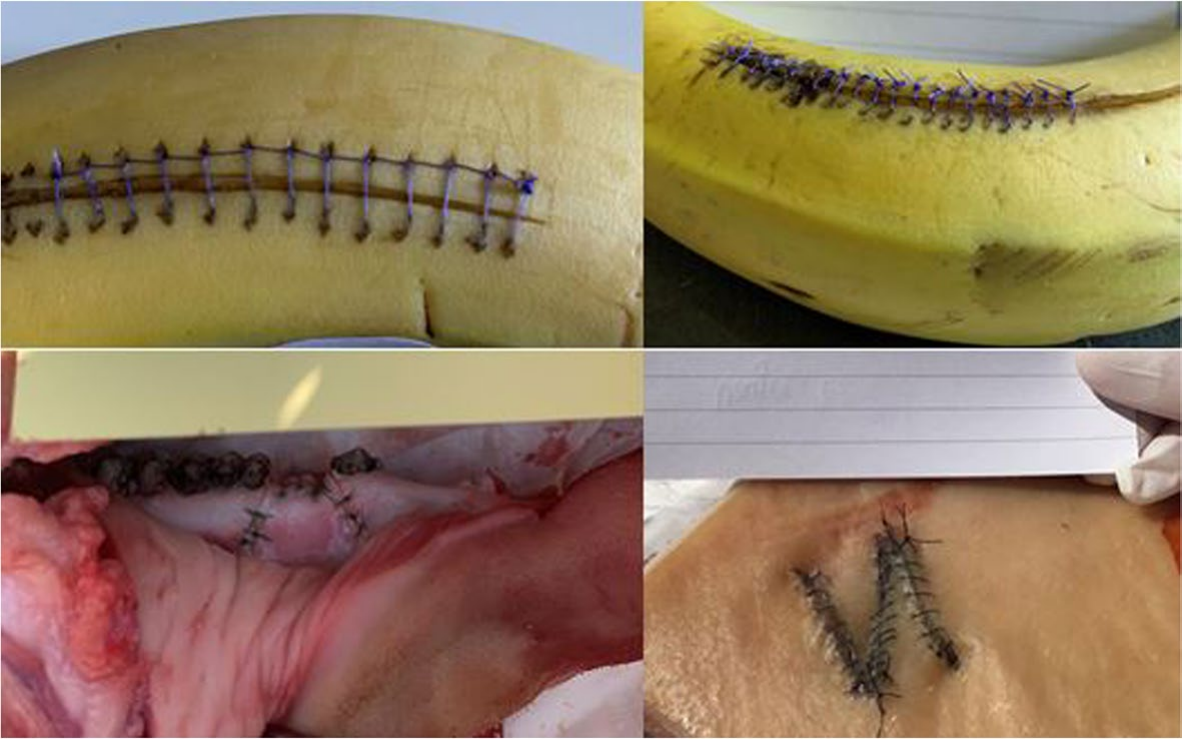
Examples of students’ results during their practical online/home sessions

Reviewing Module C (students’ photo documentation) showed satisfactory to very good practical results in its entirety (see Fig. 4 for examples). The uploaded images were not valued with marks, and the quality of the exercises performed had no influence on passing the course. The images were only used to provide photographic evidence that each student had passed each course day.

### Statistical analysis of student surveys

A total of 12 questionnaires (= 1.8%) were not included in the statistical evaluation on the basis of the aforementioned exclusion criteria. These were four questionnaires for the final course evaluation (via StudOn), which were excluded due to incompleteness (= 1.9%). In addition, with respect to self-evaluation 1 (*n* = 6; 2.5%) and self-evaluation 2 (*n* = 2; 0.9%), a total of eight (= 1.8%) other questionnaires were not included in the statistical assessment (in total, two questionnaires were excluded because of choosing answer option 1 in 100% of the questions).

Included in the statistical analysis were a total of 205 final course evaluation questionnaires (via StudOn; = 98.1%), with 231 self-evaluations at the beginning of the SOS Course (SE1; = 97.5%) and 206 self-evaluations after going through the SOS Course (SE2; = 99.0%).

#### Results of self-evaluations 1 and 2 (see Tables 3 and 4)

**Table 3:** Student self-evaluation before and after completing the SOS-Course (questions 1 to 12)

*n* = x (%)		**general surgical skills**			**special surgical skills**								
		**Q1**	**Q2**	**Q3**	**Q4**	**Q5**	**Q6**	**Q7**	**Q8**	**Q9**	**Q10**	**Q11**	**Q12**
**before**	Abstentions:	6	5	7	30	27	115	119	118	110	114	80	77
	Grade 1:	10 (4.4%)	15 (6.6%)	10 (4.5%)	11 (5.5%)	9 (4.4%)	0 (0%)	0 (0%)	0 (0%)	1 (0.8%)	0 (0%)	6 (4.0%)	4 (2.6%)
	2:	65 (28.9%)	89 (39.4%)	82 (36.8%)	53 (26.4%)	60 (29.4%)	10 (8.6%)	5 (4.5%)	6 (5.3%)	10 (8.3%)	4 (3.4%)	28 (18.5%)	30 (19.5%)
	3:	11 (49.3%)	93 (41.2%)	94 (42.2%)	75 (37.3%)	94 (46.1%)	25 (21.6%)	19 (17.1%)	18 (15.9%)	21 (17.4%)	24 (20.5%)	55 (36.4%)	60 (39.0%)
	4:	26 (11.6%)	16 (7.1%)	32 (14.3%)	41 (20.4%)	22 (10.8%)	27 (23.3%)	27 (24.3%)	28 (24.8%)	34 (28.1%)	31 (26.5%)	27 (17.9%)	25 (16.2%)
	5:	7 (3.1%)	8 (3.5%)	3 (1.3%)	14 (7.0%)	11 (5.4%)	25 (21.6%)	31 (27.9%)	31 (27.4%)	20 (16.5%)	24 (20.5%)	13 (8.6%)	14 (9.1%)
	6:	6 (2.7%)	5 (2.2%)	2 (0.9%)	7 (3.5%)	8 (3.9%)	29 (25.0%)	29 (26.1%)	30 (26.5%)	35 (28.9%)	34 (29.1%)	22 (14.6%)	21 (13.6%)
	**Median:**	**3.00**	**2.00**	**3.00**	**3.00**	**3.00**	**4.00**	**5.00**	**5.00**	**4.00**	**4.00**	**3.00**	**3.00**
	IQR:	1	1	1	2	1	3	2	2	3	2	1	1
**after**	Abstentions:	1	1	1	11	1	2	2	2	2	3	2	2
	Grade 1:	30 (14.6%)	31 (15.1%)	17 (8.3%)	24 (12.3%)	60 (29.4%)	39 (19.1%)	38 (18.8%)	44 (21.6%)	33 (16.2%)	27 (13.4%)	34 (16.7%)	40 (19.6%)
	2:	138 (67.3%)	128 (62.4%)	132 (64.4%)	80 (41.0%)	119 (58.3%)	106 (52.0%)	103 (51.0%)	107 (52.5%)	107 (52.5%)	100 (49.5%)	87 (42.6%)	103 (50.5%)
	3:	33 (16.1%)	41 (20.0%)	47 (22.9%)	75 (38.5%)	21 (10.3%)	49 (24.0%)	53 (26.2%)	47 (23.0%)	50 (24.5%)	60 (29.7%)	66 (32.4%)	53 (26.0%)
	4:	3 (1.5%)	4 (2.0%)	7 (3.4%)	13 (6.7%)	3 (1.5%)	8 (3.9%)	7 (3.5%)	4 (2.0%)	13 (6.4%)	13 (6.4%)	13 (6.4%)	6 (2.9%)
	5:	1 (0.5%)	1 (0.5%)	1 (0.5%)	3 (1.5%)	0 (0%)	2 (1.0%)	1 (0.5%)	2 (1.0%)	1 (0.5%)	2 (1.0%)	3 (1.5%)	1 (0.5%)
	6:	0 (0%)	0 (0%)	1 (0.5%)	0 (0%)	1 (0.5%)	0 (0%)	0 (0%)	0 (0%)	0 (0%)	0 (0%)	1 (0.5%)	1 (0.5%)
	**Median:**	**2.00**	**2.00**	**2.00**	**2.00**	**2.00**	**2.00**	**2.00**	**2.00**	**2.00**	**2.00**	**2.00**	**2.00**
	IQR:	0	0	1	1	1	1	1	1	1	0	0	1
**Wilcoxon signed-rank test,** ***p*** **= ** Significance level: 0.05		< 0.001	< 0.001	< 0.001	< 0.001	< 0.001	< 0.001	< 0.001	< 0.001	< 0.001	< 0.001	< 0.001	< 0.001

Q1: Handling of surgical instruments; Q2: Manual surgical skills; Q3: Theoretical skills; Q4: Ability to coach and direct others; Q5: Suturing techniques; Q6: Z-Plasty; Q7: Kite-Flap; Q8: H-Flap; Q9: Full thickness skin graft (removal and processing); Q10: Free mucosal transplant, Q11: Tooth extraction; Q12: Mucoperiosteal Flap.Grading with German school grades 1 to 6: 1 = very good (the performance meets the requirements to a particular degree), 2 = good (the performance fully meets the requirements), 3 = satisfactory (the performance generally meets the requirements), 4 = sufficient (the performance has deficiencies but still meets the requirements), 5 = poor (the performance does not meet the requirements, but the necessary basic knowledge is available and the deficiencies can be remedied in the foreseeable future), 6 = insufficient (the performance does not meet the requirements, the basic knowledge is so incomplete that deficiencies cannot be remedied in the foreseeable future). The answer “I don´t know” was counted as abstention. IQR = Inter Quartile Range

**Table 4:** Student self-evaluation before and after completing the SOS Course (questions 13 to 15)

*n* = x (%)		**Q13**	**Q14**	**Q15**
**before**	Abstentions:	8	17	60
	I absolutely agree (1):	67 (29.9%)	47 (22.0%)	6 (3.5%)
	I somewhat agree (2):	146 (65.2%)	140 (65.4%)	28 (16.4%)
	I rather disagree (3):	11(4.9%)	27 (12.6%)	112 (65.5%)
	I do not agree at all (4):	0 (0%)	0 (0%)	25 (14.6%)
	**Median:**	**2.00**	**2.00**	**3.00**
	IQR:	1	0	0
**after**	Abstentions:	3	8	49
	I absolutely agree (1):	74 (36.5%)	53 (26.9%)	6 (3.8%)
	I somewhat agree (2):	129 (63.5%)	122 (61.9%)	16 (10.3%)
	I rather disagree (3):	0 (0%)	22 (11.2%)	109 (69.9%)
	I do not agree at all (4):	0 (0%)	0 (0%)	25 (16.0%)
	**Median:**	**2.00**	**2.00**	**3.00**
	IQR:	1	1	0
**Wilcoxon signed-rank test, ** ***p*** ** = Significance level: 0.05**		.008	*.157*	< 0.001

Q13: I am convinced that I will be able to cope well with the future practical requirements (during the course/in working life)

Q14: I am rather relaxed about possible difficulties during the practical work because I can trust in my own capabilities

Q15: Others are better able to cope with future practical requirements (during the course/in working life)

The answer “I don´t know” was counted as abstention

IQR = Interquartile Range

The evaluation of the self-evaluation questionnaires and the improvement in median score showed that the general surgical skills of the students (Q1–3) improved during the course (self-assessed). The school grades used to evaluate their general practical skills (from 1 to 6), which students assigned before and after taking the course, differed highly significantly (*p* < 0.001 for questions 1, 2, and 3). For example, the median score for surgical instrument handling (Q1) and theoretical skills (Q3) improved by one grade level (from a score of 3 to a score of 2; see Table 3). The number of abstentions was low when answering the questions on general surgical skills (Q1: n(before) = 6, n(after) = 1; Q2: n(before) = 5, n(after) = 1; Q3: n(before) = 7, n(after) = 1).

The number of abstentions on questions related to specific surgical skills (questions 4–12) was between n(before) = 27 (Question 5) and n(before) = 119 (Question 7) at the beginning of the course. After completion of the course, the number of abstentions ranged from n(after) = 1 (Question 5) to n(after) = 11 (Question 4).

All questions on specific surgical techniques (Q4–12) also showed highly significant results. The median score improved after completing the course in each case concerning specific surgical techniques (*p* < 0.001 for each; see Table 3). For example, at the beginning of the course, students rated their skills in performing a kite flap as well as an H-flap plasty, each with a school grade of 5 (median). After completing the course, the median improved to a school grade of 2. The performance of a Z-plasty (Q6), a full-thickness skin graft (Q9), and a free mucosal graft (Q10) each improved by two grade levels (from a median of 4 to 2). The ability to coach and direct others (Q4), to perform a variety of suturing techniques (Q5), as well as a tooth extraction (Q11) and creation of a mucoperiosteal flap (Q12) also improved through the course, with significant results (*p* < 0.001) from median 3 to 2.

At the beginning of the course, 29.9% of the students (*n* = 67) absolutely agreed that they would be able to handle future practical requirements well (Q13). 65.2% somewhat agreed (*n* = 146) and 4.9% rather disagreed (*n* = 11) (see Table 4). The implementation of the course resulted in significantly more students (*p* = 0.008) being confident in their practical skills to meet future requirements (in working life). Most students were relaxed about possible difficulties during future practical work, both before (≙ 87.4%, *n* = 187) and after (≙ 88.8%, *n* = 175) completion of the course (Q14). Here, there was no significant difference between the data before and after implementation of the course (*p* = 0.157). At the beginning of the course, 80.1% (*n* = 137) disagreed with the statement that others were generally more able to fulfill future practical requirements (Q15). After course completion, an even more highly significant proportion (85.9%, *n* = 134) of the students held this opinion (*p* < 0.001).

#### Course evaluation results (see Tables 5 and 6)

**Table 5:** Overview of the student course evaluation (at course completion)

	**Median**	**IQR**	**Most frequent answer (%)**
Total course rating^a^	**9.00**	2	9 and 10 (each 29.8%)
Transfer/application of knowledge in professional life^a^	**9.00**	3	10 (38.5%)
Quantity of course content^b^	**5.00**	0	5 (59.5%)
Successful teaching of the learning objectives^a^	**9.00**	2	10 (34.1%)
Overall technical implementation^a^	**9.00**	3	9 (27.3%)
Quality of the images used^a^	**9.00**	3	9 (28.3%)
Quality of teaching videos used^a^	**9.00**	2	10 (29.3%)

^a^Rating on a Likert scale from 1 (extremely bad) to 10 (very good)

^b^Rating on a Likert scale from 1 (far too much) to 10 (far too little)

*IQR* Interquartile Range

**Table 6:** Comparison of theoretical knowledge before and after course completion (student self-assessment)

Theoretical knowledge of the course content^1^		
before	Abstentions:	0
	1 (extremely bad):	13 (6.3%)
	2:	19 (9.3%)
	3:	19 (9.3%)
	4:	26 (12.7%)
	5:	23 (11.2%)
	6:	22 (10.7%)
	7:	22 (10.7%)
	8:	24 (11.7%)
	9:	24 (11.7%)
	10 (very good):	13 (6.3%)
	Median:	6.00
	IQR:	5
after	Abstentions:	0
	1 (extremely bad):	1 (0.5%)
	2:	1 (0.5%)
	3:	2 (1.0%)
	4:	0 (0%)
	5:	2 (1.0%)
	6:	8 (3.9%)
	7:	28 (13.7%)
	8:	63 (30.7%)
	9:	78 (38.0%)
	10 (very good):	22 (10.7%)
	Median:	8.00
	IQR:	1
Wilcoxon signed-rank test, *p* = Significance level: 0.05		< 0.001

^1^Rating on a Likert scale from 1 (extremely bad) to 10 (very good)

*IQR* Interquartile Range

Approximately 2/3 of the students (*n* = 122; 59.6%) rated the course overall as very good/excellent, i.e., with a grade of 9 or 10 (on a Likert scale of 1 to 10, where 1 meant “extremely bad” and 10 meant “very good”). Of the students, 59.5% (*n* = 122) described the scope of the course content as exactly right. 38.5% (*n* = 79) described the applicability of the skills learned for their later professional lives as extremely good (grading = 10, median = 9). According to the survey, the previously set learning objectives were successfully conveyed (median = 9, most frequent answer = 10 with 34.1% and *n* = 70). The technical implementation of the course was rated overall with a median of 9 (Inter Quartile Range/IQR = 3). The images and video material used to illustrate the content were also rated with a median of 9 (images: IQR = 3, most frequent response = 9 with 28.3% and *n* = 58; videos: IQR = 2, most frequent answer = 10 with 29.3% and *n* = 60).

The comparison of the theoretical knowledge level between the time points before and after the completion of the SOS Course (by self-assessment of the students) showed significant differences (*p* < 0.001; see Table 6). At the median, students rated their theoretical background knowledge of the topics covered before the course at a value of 6 with a wide dispersion (IQR = 5). After completing the course, the median was significantly higher, at a value of 8, with an IQR of 1.

## Discussion

The results obtained in this study are based on self-evaluations (by the students) and provide an indication that the newly developed course is a promising concept that can teach practical skills to dental students, even in the pandemic era. In addition to theoretical content, the introduced course is primarily intended to train practical skills online, even if students cannot be present in person (on site). The course structure, the course flow, and the content as well as the technical implementation were evaluated by the students at the end of the course with the help of (self-developed) questionnaires.

The main goal of dental education should be to train independent and self-reliant dentists who can treat patients safely and effectively. In the field of dentistry, this requires, above all, fine motor skills and manual dexterity, which should be trained through practical education [2, 12]. This can be done, for example, on a model or on a patient (under supervision and guidance). Even during a pandemic, as triggered by the spread of the coronavirus, adequate (practical) training must be ensured, despite existing contact restrictions. This key requirement for ensuring the continuity and quality of dental education was also formulated in a publication by Deery et al. (2020). The use of technologies (in the sense of digital teaching) is mentioned and demanded as a solution [13].

Initial studies during the pandemic were able to show that dental students at university hospitals in Germany were very satisfied with the provision, quality, and benefits of first digital teaching concepts [14, 15].

Other studies, already conducted before the corona crisis, have also shown that online teaching and the provision of digital media have a fundamentally positive influence on students’ interest in the learning material; students seem to be generally open to e-learning courses, and this has a positive effect on learning success [14, 16, 17].

Digital teaching concepts entail some advantages but also disadvantages. On the positive side, in addition to adherence to strict COVID regulations, e-learning generally promotes self-learning skills and the ability to use online resources, according to previous studies [18, 19]. The principle applied here is constructivism, which is a recognized and widely discussed educational learning theory. It criticizes the conventional forms of pure "representational teaching." The focus is on action-oriented forms of teaching and learning. Digital media have an enormous influence here and can create a constructivist learning environment. Through multimodal learning, an individual reality can be created by using many sensory organs, and knowledge can be newly (and actively) constructed. Important for this is the independent examination of the learning content and the independent discovery of contexts [20].

The latest digital developments, such as virtual reality (VR), offer students the possibility of a realistic simulation (for example, of surgical procedures) on a virtual model through haptic technologies [2]. Here, both the student and the instructor receive integrated, continuous feedback on the student’s performance [21]. Studies have shown that the use of VR technology can improve the acquisition of skills in surgical dentistry [22].

Manual dexterity and fine motor skills can be significantly enhanced in such a simulation environment (during basic training), but this is a real challenge, as the time and resources available are not unlimited [12]. In addition, such VR systems are not portable and cannot currently be used during the pandemic [5].

As another factor related to the success of online teaching, in addition to the students’ experience with and attitudes toward online teaching, the dependence on the attitudes and interactive teaching styles of the implementing faculty has to be mentioned [23]. Faculty should have some basic knowledge of teaching and innovative technologies.

In digital teaching, a fundamental distinction between two different concepts for conveying theoretical content has to be made. Synchronous teaching is, for example, online lectures without time offset, whereas asynchronous teaching is, for example, lecture recordings and interactive online teaching material (on demand). The main difference here lies in the type of communication, direct feedback, and (time) flexibility [14]. Synchronous teaching offers the advantage of interaction with peers and encouragement of critical thinking (at a beginner’s level). Asynchronous teaching can be used to facilitate collaborative learning and is flexible in time and space. Mixed learning, consisting of both elements, can be effective in teaching integrated content and its clinical application [5, 24]. A statistical comparison of these two methods in previous publications showed that students preferred an asynchronous approach, but it also turned out that student interaction decreased significantly under asynchronous teaching [14].

Worldwide, many digital teaching concepts have been developed in the field of dental education due to the COVID-19 pandemic. The results have been widely published. Mainly, electronic platforms for e-learning are used to teach theoretical content [25]. A study by Quinn et al. [26] showed that (by April 2020) 90% of the 69 participating dental schools in Europe used online educational software tools, 72% used live or streaming video, 48% provided links to other online materials, and 65% organized virtual meetings. All of these tools were established to enable non-clinical teaching. To perform clinically based or practical teaching manikins and physical typodonts are traditionally used for the first two years of teaching before going on to patient treatment [25]. In a publication by Huth et al. [27], hands-on training on phantom heads and 3D-printed teeth continued during the pandemic. This was done in small groups within clinic rooms. In this study, 59.5% (*n* = 47 of 79 questionnaires included) considered phantom heads the best substitute for live patient care and 88.6% (*n* = 70 of 79) rated the course organization as very good/good. A decisive disadvantage from the authors´ point of view, however, is that the practical part of the course, as mentioned above, had to take place in the clinic rooms and thus under particularly elaborate protective measures [28].

In many parts of the world, practical dental training, including on phantom heads, could not continue during the pandemic because of restricted access to buildings. As a result, many schools intended to use the "closure" periods to fill the curriculum with academic activities that include online learning in the hope that students would have more time for clinics upon their return [26].

The authors sought to challenge the sole delivery of ‘theory’ and felt that postponing the teaching of practical skills was not an option – a course concept was to be created in which the practical exercises would continue (on models) without the students having to come to the clinic and thus be exposed to an increased risk of infection. In addition, of course, the theoretical content should be adequately conveyed.

To close the gap between the teaching of theoretical content and the learning of practical skills in digital online teaching, the SOS Course ("Surgical Online Skills") was developed, which includes both theoretical (synchronous and asynchronous) and practical content. To the authors’ knowledge, there is no current literature or statistical data on such an online learning concept in dentistry to date, in which the practical exercises are performed on the model at home (online).

The challenges of such a concept have already been described in the literature: online simulation on models (such as mannequins) requires significant time, personnel, and technical effort. Objective structured clinical examinations (OSCEs) have also been described as a possible evaluation method [5]. All these findings were considered when creating the course.

The results of the course evaluation by the students showed that the newly introduced course was perceived as consistently positive by the students. The statistical evaluations of the self-evaluations showed that the students rated themselves significantly better in both the theoretical and practical parts after completing the course (with a significant result). Both general surgical skills, such as the use of needle holders and forceps, and special surgical techniques, such as the formation of a mucoperiosteal flap or the correct performance of a tooth extraction, were considered to be well taught by the students. The course content was mostly considered appropriate. The authors are aware that some practical content, such as performing a kite flap or H-flap, is not necessarily part of the dental or oral surgery catalog. However, the authors believe that knowledge of the theoretical basis and performance of these specialized surgical techniques can only benefit students. By having a simultaneous positive effect on general surgical skills, it should provide students with confidence. This effect is reflected in the statistical evaluation, in which it was shown that students were relaxed about future practical demands (according to their self-evaluations).

The course evaluation with regard to the technical implementation of the course also yielded good to very good results. Over the entire course, a small technical improvement was made: a new microphone was purchased to improve the sound quality. Good image quality could be ensured through the use of high-quality cameras (acquired as part of the QuiS project; see the funding section). By using multiple cameras and a sufficient camera change, the surgical techniques could be followed from different angles and from the same perspective for each student during the pre-preparation by the lecturer.

This contrasts with traditional face-to-face classes, where a good view of the surgical site cannot always be guaranteed to every single student during pre-preparation.

Besides the consistently positive verbal feedback and the overall positive statistical data, there are some disadvantages of the course or limitations of the study that need to be discussed.

It should be mentioned, for example, that despite a high evaluation of the instructional images and videos used, an increase in the quality of the materials provided is still possible and necessary. In addition, basic spatial and technical equipment is required on the part of both the teachers and the students to conduct the course. The higher the technical effort, the better the transmission quality of the image and sound. In addition to the necessary hardware, students and teachers should have a range of "soft skills" (such as dealing adequately with other people and basic technical knowledge) and should be open to technical innovations.

Another limitation from the lecturers' point of view is that the ratio of students to tutors (compared to common practical face-to-face sessions) was very high (with 23:1 respectively 45:1), i.e. many students were connected online in relation to relatively few lecturers and tutors. This was not perceived as negative by students in the evaluation, but a smaller group of students could lead to more questions or better interactivity. Therefore, smaller groups of participants could be used in future online courses to improve course quality.

Furthermore, in future practical tests of the students, the same tasks could be set to better compare the results before and after passing the course. So far, the practical test at the beginning of the course (single button suture & horizontal mattress suture) and at the end of the course (tooth extraction & mucoperiosteal flap) did not have the same assignment. The lecturers chose different task sets because repetition through the tests alone would bring improvement in students´ skills and self-evaluations anyway. However, this makes comparability of the students' self-evaluations less meaningful. In future iterations of the course this problem could be circumvented by, for example, requiring a tooth extraction on the left side of the upper jaw at the beginning of the course and on the opposite side after finishing the course. Thus, the learning effect caused by the execution of the test itself can be minimized and the comparability of the results of the self-evaluations can be increased in the future.

In addition, the evaluation of the practical exercises was based on self-evaluations of the students that were, therefore, not completely objective. However, student self-assessment can provide an indication of learning success.

From the authors’ point of view, it is clear that patient treatment (within dental training) can in no case be completely replaced by exercises on models/mannequins. This was also not the subject of the investigations of this work. Nevertheless, surgical exercises on models improve general practical skills and prepare students for patient treatment in a certain way.

In the context of the COVIiD-19 pandemic, a quick response to the contact restrictions and the accompanying limitations in teaching was needed, especially in the area of digital teaching. It was extremely important to the authors to continue teaching practical skills during the pandemic. In addition, a certain level of quality had to be ensured. To obtain feedback from the students on both the course content and the learning success, the questionnaires used here were developed. However, these questionnaires were not piloted or assessed for validity due to the lack of time and the general exceptional situation. Therefore, we relied on already existing (internal) questionnaires, which were created based on scientific principles and served to validate previous courses in our clinic (before the pandemic). These questionnaires were modified to meet the demands of our online course. Nevertheless, this fact is, of course, a limitation of this study. Validation of the questionnaires needs to be done for future semesters, evaluations, and publications. The initial results of this study on a new digital teaching concept consisting of theoretical and practical components appear promising overall. Further research needs to be carried out to develop the concepts of blending learning and e-learning in the field of dental education.

## Conclusions

This study showed that the introduced course, which was designed to teach dental students both theoretical and practical content during the COVID-19 pandemic through a digital online teaching concept, was positively accepted by dental students. Using self-evaluations, initial results within the present limitations of this study showed that students rated themselves better than before in terms of general and specific surgical techniques after going through the course. Overall, based on the results presented here and the numerous preliminary works in the field of online teaching during the pandemic, it can be concluded that digitalization brings some advantages and advances that need to be systematically investigated and further developed in future studies.

## Data Availability

Most of the data generated or analyzed during this study are included in this published article. The remaining datasets used and/or analyzed during the current study are available from the corresponding author on reasonable request (i.e., as the subject of further research).

## Abbreviations

CE: Clinical Examination
COVID-19: Coronavirus Disease 2019
IQR: Interquartile Range
OSCE: Objective Structured Clinical Examination
Q: Question
SARS-CoV-2: Severe acute respiratory syndrome coronavirus type 2
SE1: Self-evaluation 1
SE2: Self-evaluation 2
SOS: Surgical Online Skills
VR: Virtual Reality
WHO: World Health Organization
